# Building an inclusive culture at scientific meetings: foundations for future progress

**DOI:** 10.1099/mic.0.001527

**Published:** 2025-02-06

**Authors:** Kevin Maringer, Edward Cunningham-Oakes, Ffion Lane, Kirsty L. Jones, Bruno Francesco Rodrigues de Oliveira, Aisha Baba-Dikwa, Arindam Mitra, Blanca Perez-Sepulveda, Callie R. Chappell, Guerrino Macori, I’ah Donovan-Banfield, Prerna Vohra, Rowan Casey, Roshan Nepal, C. M. Anjam Khan

**Affiliations:** 1 The Pirbright Institute, Ash Road, Pirbright, UK; 2 Department of Infection Biology and Microbiomes, Institute of Infection, Veterinary and Ecological Sciences, University of Liverpool, Liverpool, UK; 3 NIHR Health Protection Research Unit in Gastrointestinal Infections, Liverpool, UK; 4 Microbiology Society, 14-16 Meredith Street, London, UK; 5 Department of Sport and Science, School of Health, Education, Policing and Sciences, University of Staffordshire, College Road, University Quarter, Stoke-on-Trent, UK; 6 Department of Microbiology and Parasitology, Biomedical Institute, Fluminense Federal University, Niterói, Brazil; 7 Specialist Medicine Non-Clinical Research, Renal Research, Manchester Royal Infirmary, Oxford Road, Manchester, UK; 8 School of Allied and Healthcare Professions, RV University, Bengaluru, Karnataka, India; 9 Institute of Infection, Veterinary and Ecological Sciences, University of Liverpool, Liverpool, UK; 10 Department of Biology, Stanford University, Stanford, CA, USA; 11 School of Biology and Environmental Science, University College Dublin, Dublin, Ireland; 12 NIHR Health Protection Research Unit in Emerging and Zoonotic Infections, Liverpool, UK; 13 Institute of Immunology and Infection Research, School of Biological Sciences, University of Edinburgh, Charlotte Auerbach Road, Edinburgh, UK; 14 School of Medicine, Cardiff University, Cardiff, UK; 15 Agriculture and Food (Livestock and Aquaculture), The Commonwealth Scientific and Industrial Research Organisation, Hobart, TAS, Australia; 16 Biosciences Institute, Faculty of Medical Sciences, The Medical School, Newcastle University, Newcastle, UK

**Keywords:** accessibility, conferences, DEI, diversity, EDI, equality, equity, inclusivity, marginalized scientists, Microbiology Society, scientific meetings

## Abstract

Scientific meetings and conferences are crucial in knowledge dissemination, fostering collaborations, professional development and inspiring innovative research. However, their traditional structure and organization have remained largely unchanged, perpetuating barriers that continue to exclude scientists from historically marginalized backgrounds. In response, the Microbiology Society has begun its journey to address these longstanding challenges, redesigning its meetings to create a more inclusive culture and a welcoming environment for all participants.

## Introduction

Inclusivity and accessibility at scientific conferences and meetings are of paramount importance for fostering collaboration and ensuring that individuals from diverse backgrounds feel welcome and engaged [1,2]. Promoting equality, diversity and inclusion (EDI), also referred to as diversity, equality and inclusion (DEI) at conferences creates a respectful and welcoming environment. It addresses historical biases and encourages meaningful participation from historically marginalized groups through initiatives such as updated codes of conduct and inclusive policies [1,3]. In recent years, the Microbiology Society, a UK-based members’ organization and not-for-profit publisher serving an international community of microbiologists, has made significant strides in fostering an inclusive and welcoming environment at its conferences and scientific events. By implementing recommendations and thoughtful strategies to promote inclusivity, we believe our conferences are becoming more accessible and accommodating for their diverse delegates [4]. In this article, we share our personal views, lessons learnt and strategies implemented by the Microbiology Society to achieve broader inclusivity at conferences and events organized by the Society. Most of the authors of this personal view article are members of the Microbiology Society Members Panel (likely to be re-named as the EDI Committee), which represents disadvantaged and historically marginalized microbiologists and played a crucial role in driving many of the innovations described here. We take pride in these advancements, which we will continue to refine and develop. We believe these developments can serve as a starting model for other scientific societies and learned bodies. Building a more inclusive research culture is a journey, and we share here some valuable and transferable lessons learnt whilst acknowledging there is still much more work that can and should be done [5].

## A deep-seated commitment to inclusivity

The Microbiology Society’s increasing commitment to inclusivity has been evident in the formation of the Members Panel in 2022, representing members from historically marginalized backgrounds, which has enabled the consideration of a more diverse range of lived and learnt experiences [6]. The Members Panel has done considerable work in planning and implementing more comprehensive and inclusive practices at recent conferences and events. For example, at the Annual Conference 2024 in Edinburgh (UK), the panel aimed to create an environment where participants felt represented, valued and respected. The planning process began early, first including a visit to the venue to assess the facilities and provide feedback for enhancing accessibility and inclusivity.

The second aspect addressed at an early stage was acknowledging the special dietary requirements and providing inclusive menu options that accounted for the participants’ diverse dietary and religious needs [7]. An inclusive menu accounts for dietary diversity and is crucial for ensuring participant satisfaction and health outcomes in conferences. The evidence shows that thoughtful menu planning improves dietary adherence, health and overall acceptance [8,9]. In addition to dietary options, due to the conference coinciding with the final days of the month of Ramadan and the subsequent Eid celebration, the Society provided takeaway lunches for fasting delegates to address religious dietary requirements. This consideration ensured that attendees could participate without compromising their personal and/or religious beliefs and practices. We highlight that as fasting and prayer commitments during the month of Ramadan may affect the ability of Muslim attendees to fully engage with scientific content and networking opportunities, it would be preferable in the future to avoid organizing conferences during the month of Ramadan whenever possible. When scheduling conferences, it should be borne in mind that the timing of Ramadan is based on the Islamic lunar calendar rather than the Gregorian calendar [10]. Additionally, the catering teams worked closely with the organizers to accommodate diverse dietary requirements, including vegan, dairy-/gluten-free, kosher and halal options [7,9]. Furthermore, dietary allergen lists were made widely accessible at food stations.

## Dedicated spaces for delegates with accessibility or additional needs

**Fig. 1.**
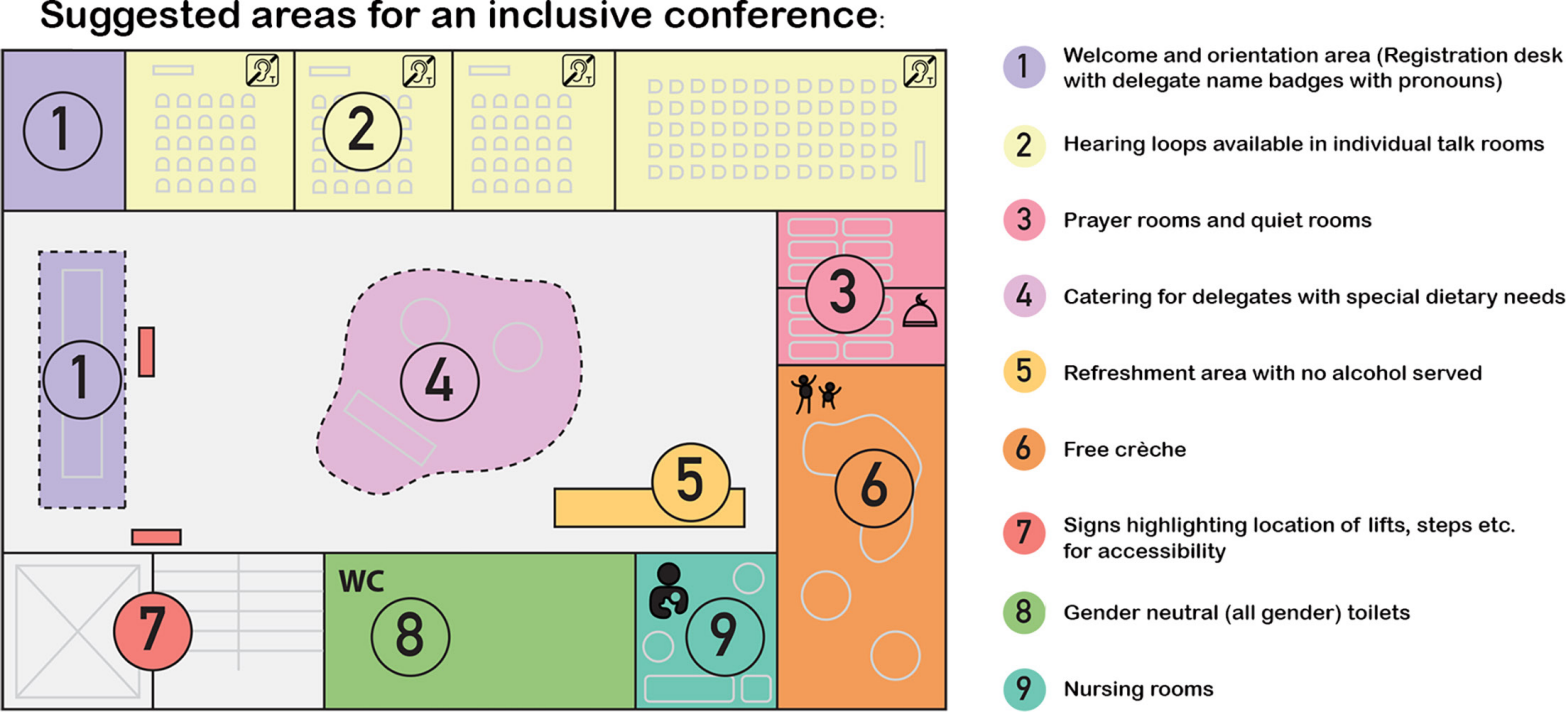
Facilities and initiatives to enhance inclusiveness at scientific conferences. The figure illustrates key facilities and initiatives aimed at fostering inclusiveness at scientific conferences. The map is indicative only and not to scale. (1) The welcome and orientation area is a key area for registration, signposting and providing delegates with name badges that include pronouns. New and international delegates can be further supported through dedicated events and orientation areas. (2) Seminar rooms should be fitted with hearing loops, and chair briefings should include information on phonetic name spellings for scheduled speakers. (3) Non-denominational, gender-segregated prayer rooms with washing facilities and prayer mats, and quiet rooms should be provided, ideally in quieter areas of the venue with lower footfall. (4) Catering should consider special dietary requirements, including the provision of takeaway boxes for fasting delegates. (5) Consideration should be given to the negative impacts of providing free alcohol, especially at scientific sessions such as poster sessions. (6) A free crèche should be provided to support delegates with young children. For child protection reasons, a space at the free crèche at Microbiology Society conferences is provided by prior appointment only and the location is disclosed only to parents after a space is confirmed. Additional funds can be made available to those delegates unable to make use of the crèche. (7) Clear signposting to accessibility and inclusivity features should be provided throughout the conference venue and be made available ahead of time (including accessibility information and sensory maps). (8) Access to dedicated gender-neutral (all gender) toilets should be provided even if not available by default at the venue. (9) A nursing room should be provided. In addition to the facilities and initiatives shown here, the main scientific programme can be enhanced through the provision of social and networking events aimed at supporting LGBTQIA+, disabled, neurodivergent and other historically marginalized groups. An equality checklist, a flexible supplementary budget, planning in advance with accessibility in mind and engaging with expert organizations leading in inclusivity is crucial for the success of an inclusive event.

It is key to consider accessibility as early as the point of selecting a venue, particularly in regions where accessibility measures are not ensured by legislation. The provision of dedicated spaces, such as quiet rooms, gender-segregated prayer rooms with washing facilities and prayer mats, as well as a nursing room, also underscored the Society’s dedication to accommodating diverse cultural and accessibility needs (Fig. 1). These spaces were available to anyone who required them, fostering an atmosphere of respect and support. Furthermore, a commercial collaboration to offer a free crèche for the children of delegates highlighted the Society’s commitment to making the conference more accessible to parents of young children. This service was available on a first-come, first-served basis and catered to children aged 0–12 years old, providing a safe and engaging environment for accompanying young attendees. Furthermore, Society Event Grants include a contribution to help with caring costs for attendees who cannot use the crèche or have other caring responsibilities [11].

Further, the Society ensured that the venue had level access routes and wheelchair-accessible toilets; hearing loops were provided in the main auditorium and other rooms upon request. These features were crucial for delegates with accessibility needs, ensuring they could navigate the conference without hindrance. The Society’s focus on inclusivity was also reflected in the commitment to providing gender-neutral toilets, which are also referred to as ‘all gender’ or ‘inclusive toilets/restrooms’, at all events organized by the Society. At the Society’s Annual Conference in 2024, gender-neutral toilets were provided as standard by the venue; however, in previous years, the conference organizers installed temporary labels to designate a subset of bathroom facilities as gender-neutral. The most straightforward approach is often to designate accessible toilets, which by design are gender-neutral, single-cubicle facilities, as gender-neutral. Accessibility information should also be provided to delegates ahead of time, if not already provided on the venue website; this should include both physical access information as well as sensory maps.

## Inclusive social events and alcohol-free sessions

The Society’s approach to social events has also evolved to be more inclusive by continuously seeking feedback and incorporating the needs or preferences of the target communities. For example, at the Microbiology Society Annual Conference 2022, Belfast (UK), numerous suggestions around neuroinclusivity, quiet spaces and alcohol-free socials were received. One attendee suggested a designated quiet room with dimmed lights for neurodivergent people, separate from the religious prayer rooms, with proper advertisement and a clear description of the room location, which would be beneficial and important. Another attendee asked the Society to ‘improve the number of seating options and include a quiet space to escape the noise and traffic of break times’, while another suggestion was to have ‘a selection of social events that include quieter, more relaxed venues with less of an emphasis on alcohol’.

While networking events or socials with alcohol are widely accepted in Western and European culture, some perceive the provision of alcohol at scientific conferences as problematic, as it is not inclusive of people with cultural traditions that avoid alcohol consumption or people who lead a sober lifestyle [12]. Additionally*,* overindulgence in alcohol could lead to an increase in antisocial behaviour, inappropriate comments and sexual harassment [7, 13]. Furthermore, early career researchers have reported that turning a scientific poster session into an alcohol-fuelled social event gives the impression that work presented by early career researchers at poster sessions is of lesser value [13,14]. Recognizing the diverse preferences of the attendees and the feedback received, alcohol was removed from the scientific poster session area at the Microbiology Society Annual Conference 2023. This change aimed to ensure everyone could engage fully without discomfort. However, acknowledging mixed feedback following the complete removal of alcohol from the conference in 2023, a drinks reception was reintroduced for those who wished to enjoy alcoholic beverages in a designated setting during the Microbiology Society Annual Conference 2024, with alcohol-free options available [12]. Additionally, the continuation of an LGBTQIA+ social event, organized by a group of members, self-named ‘Queer-in-Micro’, now in its third year and the introduction of the Disabled and Neurodivergent Members Social, reflected a proactive stance in creating networking opportunities for people from historically marginalized backgrounds [15]. These events provided safe and supportive environments specific for diverse communities to connect and share their experiences.

Neuroinclusivity in scientific meetings can be promoted by implementing accommodations such as quiet spaces to reduce sensory overload, dietary options to address sensory and nutritional sensitivities and virtual attendance to enhance accessibility. These measures mitigate sensory and social challenges, fostering equitable participation for neurodiverse individuals [16]. For example, extensive prior communication was provided for the Disabled and Neurodivergent Members Social to alleviate apprehensions delegates may have regarding accessibility needs (both physical and sensory) being met [16]. The social, for example, provided sensory maps ahead of time, as well as a quiet room, activities and fidget toys during the event, while making it clear that delegates with specific sensitivities around food were allowed to provide their own (with prior agreement by the caterer). Clarity was also provided that the social was inclusive to all who identify as neurodivergent or disabled, regardless of official diagnosis. Clear communication is valued, particularly in an international setting, as definitions of and information on disability and neurodivergence can differ between countries. A designated meeting place for conference delegates to make their way to the social can reduce apprehension and further encourage attendance. Both socials also had clear policies on the taking and sharing of photos to respect attendees’ right to privacy and create a safe space. In addition, both socials were alcohol-free.

## Supporting new and international delegates

To assist newcomers and international delegates, the Society offered a tour of the venue and meet-up opportunities during the conference. These initiatives helped first-time attendees and international delegates to break the ice and feel welcomed into the conference and community. The meet-up events provided a platform for international delegates to network, share their unique perspectives and foster cross-cultural collaborations. Society staff were available to answer questions and guide new delegates throughout the conference, ensuring a smooth and enjoyable experience.

## The power of names: ensuring accurate pronunciation, spelling and pronoun use

Inclusivity at conferences also extends to how we address each other. Using correct name pronunciations and spellings as well as pronouns ensures that everyone feels seen and valued [17]. This practice was encouraged at the conference and helped to create an environment where all participants, regardless of their background, felt included and respected. A positive change this year was that delegates were encouraged to provide their preferred pronouns during the registration, allowing these to be printed directly onto name badges. This initiative increased the number of delegates specifying their pronouns over previous years, where name badges simply provided a ‘blank’ space for delegates to add their pronouns by hand. Moreover, abstract submission forms also asked for pronouns and name pronunciation, which were shared with session chairs to ensure respectful and accurate addressing of the speakers. Phonetic spelling was included in speakers lists provided to session chairs, which, over the years, has included increasing guidance to chairs and delegates, standardizing the approach to phonetic spelling and increasing the success of this initiative. In addition, there are digital tools available to help pronounce names correctly.

## Equality, diversity and inclusion checklist

To ensure consistency and institutional memory of lessons learnt, the Microbiology Society’s comprehensive EDI checklist for events ensures that all venues and resources are as accessible and inclusive as possible (Fig. 1). This checklist covers essentials such as accessible/gender-neutral toilets, hearing loops, level access and prayer/quiet rooms. It also includes additional considerations for larger events, such as audio description, braille options and a crèche. By adhering to these guidelines, the Microbiology Society sets a standard for other organizations to follow. The checklist also emphasises the importance of clear communication about accessibility features on event webpages and emails, ensuring that attendees are well-informed about the available options and facilities.

The Microbiology Society creates awareness of EDI issues via its publications and by organizing an EDI session at the Society’s Annual Conference. The Microbiology Society is now a member of Equality, Diversity and Inclusion in Science and Health (EDIS), which is a coalition of organizations working to improve EDI within the science and health research sector [18]. Being a member of EDIS allows the Society to gain and share knowledge, recommendations, lessons learnt and best practices in EDI with other similar organizations [19].

## An evolving journey

The improvements made to Microbiology Society conferences reflect an increasing commitment to creating an inclusive and welcoming environment for all delegates so that everyone can comfortably participate in these valuable events without significant hindrances. Moreover, by actively seeking feedback and making continuous improvements, the Society demonstrates its dedication to inclusivity. For example, following community engagement, additional social and networking events to support trans and nonbinary delegates were introduced, which is particularly in line with a recent perspective on building more queer and trans-inclusive microbiology conferences [20].

Communication and signposting have also been flagged as an area for improvement, ensuring that the right information reaches the right delegates through accessible channels. We are on an ongoing journey to simplify and improve name pronunciation and increase the sharing of pronouns by event delegates. To improve this, session chairs should be further supported to ensure they are fully familiar with the correct pronunciation of speakers’ names before making introductions. Dedicated budgets to meet unforeseen needs, such as sign language interpreters if needed, can help organizers be responsive to delegates’ needs later in the organizational process after the overall budget has been set. It should also be acknowledged that organizations often work within the constraints of the venue. While some accommodations can almost always be made (for example, the provision of gender-neutral toilets), others can be more challenging (for instance, reducing sensory stimulation during poster sessions to increase accessibility for neurodivergent delegates), and, therefore, time needs to be dedicated to community engagement and proactive problem-solving. Seeking out the increasingly available existing resources, for example, on providing LGBTQIA+ inclusive international conferences, is a good starting point for event organizers [21].

## Concluding reflections

Great gains can be made by seeing inclusivity as an integral part of conference organization, rather than a last-minute annex. The Members Panel introduced an EDI session at the Microbiology Society’s Annual Conference, now in its second year, which uses the visibility and large attendance to share challenges faced and lessons learnt regarding improving inclusivity across the sector. This year’s (2024) focus was on ‘Cultivating diversity: practical ways to make microbiology more inclusive’ with speakers showing examples of actions that are contributing to ‘making the field of microbiology more welcoming and inclusive to all’.

As these initiatives continue to evolve, they can serve as a starting blueprint to build upon for other academic societies and communities to foster a culture of inclusivity and respect across the broader scientific landscape. By prioritizing EDI considerations, we can build a more diverse, equitable and inclusive community for all scientists. Our commitment to continuous improvement emphasises that our current approach is just the starting point, allowing us to evolve and ensure a better environment for everyone.

## Abbreviations

DEI: diversity, equality and inclusion
EDI: equality, diversity and inclusion
EDIS: Equality, Diversity and Inclusion in Science and Health
